# Quality of life in flexible painful flatfoot treated by anterograde calcaneo-stop procedure: The patient’s and family’s perspective

**DOI:** 10.1371/journal.pone.0263763

**Published:** 2023-02-02

**Authors:** Daniela Dibello, Gabriele Dallan, Valentina Di Carlo, Federica Pederiva

**Affiliations:** 1 Pediatric Orthopedics, Institute for Maternal and Child Health—IRCCS “Burlo Garofolo”–Trieste, Italy; 2 University of Trieste, Trieste, Italy; 3 Pediatric Surgery, Institute for Maternal and Child Health—IRCCS “Burlo Garofolo”–Trieste, Italy; Policlinico A Gemelli: Fondazione Policlinico Universitario Agostino Gemelli IRCCS, ITALY

## Abstract

**Purpose:**

This study aimed to evaluate the quality of life and satisfaction about the surgical treatment in patients with symptomatic flexible flatfoot.

**Methods:**

The Oxford Ankle Foot Questionnaire for children (one to fill in before the surgical correction and another 6–12 months after the screw’s removal), the PedsQL^TM^ Healthcare Satisfaction Generic Module and the PedsQL^TM^ General Well-Being Scale were administered to all patients who underwent the anterograde calcaneo-stop procedure for flexible painful flatfoot between January 2012 and December 2015.

**Results:**

One hundred forty patients were sent the questionnaires and 74 (40 male and 34 female) of them responded. The surgical correction was performed at a medium age of 11,84±1,65 years.

When the Oxford Ankle Foot Questionnaire for children scores before surgical correction and after the screw removal were compared, the latter scored significantly higher for all domains. Healthcare satisfaction was good in all families. Most of the patients scored medium-high on the PedQLTM General Well-Being both when asked about themselves (mean 86,50±7,44) and in general about their health (76,06±12,32).

**Conclusion:**

Our results confirmed that flexible painful flatfoot is significantly affecting the quality of life of children and that the anterograde calcaneo-stop procedure is a valuable technique, which improves their quality of life and the family wellbeing.

## Introduction

Flexible flatfoot is one of the major cause of visits in the outpatient clinic for pediatric foot problems [1]. Flatfoot is a progressive acquired or developmental deformity characterized by the flattening of the medial arch, the plantar and medial rotation of the talus and the forefoot abduction [2]. Flexible flatfoot presented a normal arch during nonweightbearing and a flattening of the arch on stance. Although flatfoot rarely leads to disability, it is still a great concern for parents, and it is believed to cause gait disorders later.

Whereas this condition is sometimes asymptomatic, it can also cause pain, difficulty walking, and physical impairment. The treatment of flatfoot deformity includes nonsurgical and surgical procedures. Nonsurgical treatment consists of activity modifications, stretching, supportive footwear with medial arch supports, ortheses, mild analgesics or non-steroidal anti-inflammatory drugs. Sometimes insoles could give relief from pain [3]. Some authors have reported beneficial effects from insoles and foot exercises [4]. However, a recent systematic review has demonstrated that most nonoperative treatments have limited efficacy [5]. The purpose of surgery is to restore and maintain physiological alignment between the talus and calcaneus, allowing the foot bones to remodel themselves during the subsequent period of growth.

Among the different types of surgical treatments, we prefer to perform the anterograde calcaneo-stop procedure [6] described by Castaman. As other investigators [6–8], we believe that beyond an obvious mechanical effect, the calcaneo-stop procedure has a proprioceptive function.

Whereas many studies reported the clinical outcomes of symptomatic flexible flatfoot surgical correction [7,9,10], the quality of life of these children has not been investigated.

This study aimed to provide a comprehensive evaluation of quality of life and satisfaction on the surgical treatment in patients with symptomatic flexible flatfoot.

## Methods

After ethical approval by the Institutional Research Committee of the Institute for Maternal and Child Health—IRCCS “Burlo Garofolo” (Trieste, Italy), the Oxford Ankle Foot Questionnaire for children, the PedsQL^TM^ Healthcare Satisfaction Generic Module and the PedsQL^TM^ General Well-Being Scale were administered to a cohort of patients who were treated with arthroereisis, at our institution, for flexible painful flatfoot between January 2012 and December 2015. A written informed consent was obtained from parents of the minors included in the study. Exclusion criteria for the study were syndromes or neuromuscular disorders, congenital or post-traumatic flatfoot, postoperative complication and associated surgical times, as Achilles tendon lengthening and reduction of the appearance of scaphoid or tension of tibialis posterior tendon. We didn’t treat patients younger than 8 years old or older than 18.

An explanation of the study and the confidentiality of the data were included.

Four questionnaires were considered: the Oxford Ankle Foot Questionnaire for children, one to be filled in before the surgical correction and another 6–12 months after the screw’s removal (24–26 months after the first surgical procedure), together with the PedsQL^TM^ Healthcare Satisfaction Generic Module and the PedsQL^TM^ General Well-Being Scale.

The questionnaires were provided by Oxford University Innovation and the Mapi Research Trust (http://mapigroup.com/services/mapi-research-trust/questionnaire-distribution/) and used under their agreement.

The Oxford Ankle Foot Questionnaire includes 15 items, 14 of which are grouped into three subscales: physical (6 items), emotional (4 items), and school and play (4 items). The response options to each item are on a 5-point scale rated from never (4), rarely (3), sometimes (2), very often (1) to always (0), where the number in brackets represents the value that should be applied by the scorer to each response. The last item provides information about patient’s satisfaction on the footwear [11–13].

The PedsQL^TM^ Healthcare Satisfaction Generic Module comprises 6 domains associated with healthcare satisfaction, information, inclusion of family, communication, technical skills, emotional needs and overall satisfaction [14]. PedsQL^TM^ General Well-Being Scale is composed of 6 items, and one overall general health item.

All items were reversed-scored and linearly transformed to a 0–100 scale. The higher scores indicate better functioning and a less negative impact. Missing items were handled according to the developer’s published guidelines [15].

### Surgical technique

Surgical correction was recommended in presence of grade 3 symptomatic flexible flatfoot, defined by clinical and radiographic evaluation.

All patients with painful flexible flatfoot underwent the anterograde calcaneo-stop procedure [6] performed with local anesthesia, sometimes associated with a mild sedation, without the use of tourniquet. The patient was placed supine with the involved extremity slightly rotated inwards and a 90° bended knee. The supinated foot was kept by the assistant leaning on the fluoroscopic machine to allow clear fluoroscopic control. An incision of 1 cm was applied centered on the sinus tarsi. The astragalus was drilled and a steel screw (VCA, Mikai®, Genoa, Italy) of desired diameter and length (6.5 mm x 35 mm or 8 mm x 40 mm) was inserted at 35° direction in the sagittal and 45° in the coronal plane. The dorsiflexion of the foot was checked with the knee in extended position. No cast immobilization was required. Patients were allowed for full weight bearing the same day of the surgery or as soon as possible. Foot exercises were recommended post-operatively. The removal of the screw was scheduled 24–26 months after the first surgical procedure.

### Statistical analysis

Oxford Ankle Foot Questionnaire scores were computed in the same cohort of patients at two different endpoints, before surgical treatment and after the screw’s removal, using independent sample t-test.

The PedsQL^TM^ Healthcare Satisfaction Generic Module and the PedsQL^TM^ General Well-Being Scale scores were analyzed qualitatively.

Statistical analyses were conducted using the Software GraphPad Prism 6.0 and *p* value <0.05 was considered statistically significant.

## Results

From January 2012 to December 2015, we performed 500 anterograde calcaneo-stop procedures on 250 children with symptomatic flexible flatfoot. All patients underwent bilateral surgical correction.

One hundred forty patients were eligible for the study and received the questionnaires. Seventy-four patients (34 female and 40 male) responded. The overall return rate was 52.9%. All of them presented with grade 3 symptomatic flexible flatfoot, underwent surgical correction at the mean age of 11.84 ± 1.65 years and received the questionnaires at the mean age of 15.50 ± 1.76 years (**Table 1**). The degree of correction in all patients was maintained between 19 and 21. The screw was removed after 24–26 months. The cohort of patients resulted, therefore, quite homogeneous by the point of view of the clinical condition, age at surgery, degree of surgical correction and age at which the questionnaires were given. The patients were followed up in the outpatient clinic at 1, 6, 12 and 24 months after the anterograde calcaneo-stop procedure. They were seen 6 and 12 months after the screw’s removal and then once a year till the end of growth. All the patients had a successful surgical correction, with no relapse of the condition. They had no complications, no wound infections and all of them were able to return to physical activities 3 months after the surgical correction and then 1 months after the screw’s removal. All of them had a normal range of motion of the ankle joint with an improved walking speed.

**Table 1:** Demographic data, age at surgery, time between surgeries and length of follow up.

Sex			
	Male	40	55.60%
	Female	34	44.40%
Age (years)			
	Mean±SD	15.50 ± 1.76	
	Minimum	12	
	Maximum	20	
Age at surgery (years)			
	Mean±SD	11.84 ± 1.65	
	Minimum	8	
	Maximum	15	
Time between surgeries (years)			
	Mean±SD	1.99 ± 0.59	
Follow-up (years)			
	Mean±SD	3.18 ± 0.98	

The questionnaires were given 6–12 months after the screw’s removal, when the wound was considered stabilized.

When the disability associated with flatfoot was investigated, it resulted that there were physical limitations in terms of having difficulties when walking, running or standing for long time, emotional involvement, which affected the ability to socialize with peers, and footwear problems before treatment. All the items significantly improved after the surgical correction of flatfoot (**Table 2**).

**Table 2:** Scale descriptives for Oxford Ankle Foot Questionnaire for children: Comparison across time before surgical correction and after the screw removal.

Dimension	Baseline		Follow-up		Difference	*p* value
	Mean	SD	Mean	SD		
Physical	64.67	13.69	80.99	10.67	16.31	<0.001
School and play	83.10	8.03	91.29	6.54	8.19	<0.01
Emotional	77.55	12.15	93.40	4.57	15.85	<0.001
Footwear	59.51	30.36	86.27	23.66	26.76	<0.001

The healthcare satisfaction was good in all families (**Table 3**). Most of the patients scored medium-high on the PedQL^TM^ General Well-Being (**Table 4**) both when asked about themselves (mean 86,50±7,44) and in general about their health (76,06±12,32).

**Table 3:** Scale descriptives for PedsQL ^TM^ Healthcare Satisfaction Generic Module.

PedsQL HealthCare Satisfaction		
	Mean	SD
Information	90.56	6.88
Inclusion of Family	92.54	4.73
Communication	93.24	4.27
Technical Skills	92.00	3.90
Emotional Needs	86.64	4.48
Overall Satisfaction	95.05	2.98

**Table 4:** Scale descriptives for PedsQL ^TM^ 4.0 Generic Core Scales.

PedsQL General Well-Being Scale		
	Mean	SD
About me	86.50	7.44
In general	76.06	12.32

## Discussion

Flexible flatfoot is a common condition in the childhood. It has recently been demonstrated that musculoskeletal problems involving the foot were the most common reason for calling a consultation [16] and flatfoot was the most frequent condition seen in pediatric orthopedic clinic [17]. The surgical treatment of flatfoot is foreseen in cases in which conservative management has not led to improvements, especially when it is associated with painful symptoms, early fatigue and motor hindrance. Among the surgical procedures to correct symptomatic flexible flatfoot, our choice is the anterograde calcaneo-stop procedure [6] described by Castaman. We share the opinion of other authors [6–8] that, in addition to the mechanical action of the bone repositioning, the screw maintain the correction by stimulating the proprioceptive receptors around the sinus tarsi [18].

The clinical outcomes of this procedure have been described by many authors [7,9,10]. However, the quality of life of these children before and after surgery has not been extensively investigated. We therefore evaluated the quality of life and satisfaction on the surgical treatment in patients with symptomatic flexible flatfoot using the Oxford Ankle Foot Questionnaire for children. When physical function in patients with symptomatic flexible flatfoot was concerned, the results of the questionnaires highlighted difficulties in walking or running, with soreness or aching, and foot and ankle pain before surgery. Moreover, the condition seemed to affect the possibility and the willing of the patients to go play with friends, either at school or at after school outings, or to take part in PE lessons. The situation had an impact on the emotional side of the patients, who had been bothered by their way of walking and by the appearance of their foot and who had been forced to wear different shoes from the ones they would have liked. Inevitably, this burdened the parents of the patients, who were preoccupied because their children avoided socializing with peers and spent more time alone at home, feeling different from their friends. The parents were also troubled in finding ways to entertain their sons and daughters without the help of the sport activities, which would have been good for a healthy physical and mental growth. The more they tried to make their children feel equal to their peers, the less they succeeded, considering that most of the children were also facing the troubles of adolescence. The topic of the shoes was another occasion of confrontation, as it was hard to find something their children like to wear without feeling weird or being bullied by their schoolmates.

All items significantly improved after the surgical correction allowing the patients to walk and run easily and stand for long periods without experiencing pain. They felt free to attend leisure activities with friends without limitations and improved their self-esteem.

Looking into the psychological wellness of the patients, the PedsQL^TM^ General Well-Being Scale emphasized good health with adequate support from parents and good future expectations. At the same time, both patients and their families were highly satisfied with the care received in the hospital by the staff during the whole course of treatment. They both felt listened by the staff and comfort in their concerns.

The study has some limitations that should be acknowledged. In the first place, the parental and patient perception of the general status was subjective. It might, therefore, had been influenced by several personal factors and it might not always have matched the objective professional assessment. However, all patients were included without specific selection, except for the exclusion criteria, and therefore we believe that our cohort was most likely representative and homogeneous by the point of view of the clinical condition, age at surgery, degree of surgical correction and age at which the questionnaires were administered. Second, we acknowledge the lack of a control group, which would have helped demonstrate that after the surgery the patients regained a normal life. On the other hand, we think that the high scores of all items of the Oxford Ankle Foot Questionnaire administered after surgery might compensate for this lack.

In conclusion, our results confirmed that flexible painful flatfoot is significantly affecting the quality of life of children and that the anterograde calcaneo-stop procedure is a valuable technique, which improves their quality of life and the family wellbeing.

## Supporting information

S1 Dataset (XLSX)
